# Supplementary material for: Discovery and application of insertion-deletion (INDEL) polymorphisms for QTL mapping of early life-history traits in Atlantic salmon
Source: BMC Genomics. 2010 Mar 8;11:156. doi: 10.1186/1471-2164-11-156 (PMC2838853; doi:10.1186/1471-2164-11-156)
Supplement: Additional file 2 — Information on developed 76 locus single-run INDEL panel in Atlantic salmon. Information on fluorescence labeling, primer concentrations, PCR pooling and links to alignments, INDEL motifs and GENESCAN (Burge and Karlin 1997) predictions of genes/exons are available in html format. [file 1471-2164-11-156-S2.ZIP › Additionalfile2/table16730.html]

Contig Alignment

```
Base	A B C D E F   
1	A . . . . .    
2	G . . . . .    
3	A . . . . .    
4	T . . . . .    
5	A . . . . .    
6	G . . . . .    
7	C . . . . .    
8	A . . . . .    
9	C . . . . .    
10	C . . . . .    
11	T . . . . .    
12	T . . . . .    
13	T . . . . .    
14	T . . . . .    
15	T . . . . .    
16	T . . . . .    
17	T . . . . .    
18	C . . . . .    
19	T . . . . .    
20	A . . . . .    
21	A . . . . .    
22	C . . . . .    
23	A . . . . .    
24	A . . . . .    
25	T . . . . .    
26	G . . . . .    
27	T . . . . .    
28	A . . . . .    
29	T . . . . .    
30	A . . . . .    
31	A . . . . .    
32	G . . . . .    
33	A . . . . .    
34	G . . . . .    
35	G . . . . .    
36	G . . . . .    
37	A . . . . .    
38	G . . . . .    
39	A . . . . .    
40	T . . . . .    
41	G . . . . .    
42	A . . . . .    
43	T . . . . .    
44	G . . . . .    
45	T . . . . .    
46	A . . . . .    
47	A . . . . .    
48	T . . . . .    
49	C . . . . .    
50	C . . . . .    
51	A . . . . .    
52	A . . . . .    
53	G . . . . .    
54	C . . . . .    
55	G . . . . .    
56	G . . . . .    
57	G . . . . .    
58	C . . . . .    
59	A . . . . .    
60	T . . . . .    
61	G . . . . .    
62	T . . . . .    
63	C C . . . .    
64	A A . . . .    
65	T T . . . .    
66	T T . . . .    
67	A A . . . .    
68	T T . . . .    
69	G G . . . .    
70	A A . . . .    
71	G G . . . .    
72	C C . . . .    
73	T T . . . .    
74	T T . . . .    
75	C C . . . .    
76	A A . . . .    
77	C C . . . .    
78	C C . . . .    
79	T T . . . .    
80	G G . . . .    
81	A A . . . .    
82	G G . . . .    
83	T T . . . .    
84	T T . . . .    
85	C C . . . .    
86	T T . . . .    
87	G G . . . .    
88	A A . . . .    
89	T T . . . .    
90	T T . . . .    
91	C C . . . .    
92	C C . . . .    
93	T T . . . .    
94	T T . . . .    
95	C C . . . .    
96	A A . . . .    
97	G G . . . .    
98	T T . . . .    
99	A A . . . .    
100	T T . . . .    
101	G G . . . .    
102	C C . . . .    
103	C C . . . .    
104	A A . . . .    
105	A A . . . .    
106	C C . . . .    
107	T T . . . .    
108	A A . . . .    
109	G G . . . .    
110	C C . . . .    
111	C C . . . .    
112	A A . . . .    
113	T T . . . .    
114	T T . . . .    
115	T T . . . .    
116	A A . . . .    
117	A A . . . .    
118	T T . . . .    
119	G G . . . .    
120	G G . . . .    
121	T T . . . .    
122	T T . . . .    
123	A A . . . .    
124	A A . . . .    
125	C C . . . .    
126	T T . . . .    
127	G G . . . .    
128	A A . . . .    
129	C C . . . .    
130	A A . . . .    
131	T T . . . .    
132	A A . . . .    
133	C C . . . .    
134	G G . . . .    
135	T T . . . .    
136	T T . . . .    
137	C C . . . .    
138	A A . . . .    
139	A A . . . .    
140	G G . . . .    
141	C C . . . .    
142	G G . . . .    
143	T T . . . .    
144	G G . . . .    
145	T T . . . .    
146	C C . . . .    
147	T T . . . .    
148	G G . . . .    
149	T T . . . .    
150	C C . . . .    
151	T T . . . .    
152	A A . . . .    
153	C C . . . .    
154	T T T . . .    
155	G G G . . .    
156	T T T . . .    
157	A A A . . .    
158	G G G . . .    
159	C C C . . .    
160	A A A . . .    
161	A A A . . .    
162	T T T . . .    
163	G G G . . .    
164	C C C . . .    
165	T T T . . .    
166	A A A . . .    
167	T T T . . .    
168	G G G . . .    
169	T T T . . .    
170	A A A . . .    
171	A A A . . .    
172	T T T . . .    
173	G G G . . .    
174	G G G . . .    
175	A A A . . .    
176	T T T . . .    
177	G G G . . .    
178	C C C . . .    
179	T T T . . .    
180	G G G . . .    
181	A A A . . .    
182	A A A . . .    
183	T T T . . .    
184	G G G . . .    
185	A A A . . .    
186	C C C . . .    
187	A A A . . .    
188	G G G . . .    
189	T T T . . .    
190	G G G . . .    
191	T T T . . .    
192	T T T . . .    
193	G G G . . .    
194	G G G . . .    
195	A A A . . .    
196	A A A . . .    
197	C C C . . .    
198	C C C . . .    
199	T T T . . .    
200	A A A . . .    
201	A A A . . .    
202	A A A . . .    
203	T T T . . .    
204	G G G . . .    
205	T T T . . .    
206	G G G . . .    
207	A A A . . .    
208	A A A . . .    
209	A A A . . .    
210	G G G . . .    
211	T T T . . .    
212	T T T . . .    
213	T T T . . .    
214	G G G . . .    
215	A A A . . .    
216	G G G . . .    
217	T T T . . .    
218	G G G . . .    
219	A A A . . .    
220	T T T . . .    
221	T T T . . .    
222	C C C . . .    
223	T T T . . .    
224	G G G . . .    
225	T T T . . .    
226	G G G . . .    
227	A A A . . .    
228	T T T . . .    
229	G G G . . .    
230	G G G . . .    
231	T T T . . .    
232	A A A . . .    
233	A A A . . .    
234	T T T . . .    
235	T T T . . .    
236	G G G . . .    
237	G G G . . .    
238	T T T . . .    
239	A A A . . .    
240	T T T . . .    
241	G G G . . .    
242	T T T . . .    
243	T T T . . .    
244	G G G . . .    
245	G G G . . .    
246	T T T . . .    
247	G G G . . .    
248	T T T . . .    
249	C C C . . .    
250	C C C . . .    
251	G G G . . .    
252	A A A . . .    
253	A A A . . .    
254	G G G . . .    
255	A A A . . .    
256	G G G . . .    
257	A A A . . .    
258	A A A . . .    
259	G G G . . .    
260	T T T . . .    
261	A A A . . .    
262	A A A . . .    
263	A A A . . .    
264	T T T . . .    
265	G G G . . .    
266	T T T . . .    
267	T T T . . .    
268	T T T . . .    
269	C C C . . .    
270	C C C . . .    
271	C C C . . .    
272	C C C . . .    
273	C C C . . .    
274	A C C . . .    
275	T T T T T .    
276	T T T T T .    
277	T T T T T .    
278	T T T T T .    
279	T T T T T .    
280	T T T T T .    
281	T T T T T .    
282	A A A A A .    
283	A A A A A .    
284	A A A A A .    
285	T T T T T .    
286	A A A A A .    
287	T T T T T .    
288	A A A A A .    
289	T T T T T .    
290	A A A A A .    
291	T T T T T .    
292	G G G G G .    
293	T T T T T .    
294	T T T T T .    
295	T T T T T .    
296	T T T T T .    
297	G G G G G .    
298	G G G G G .    
299	T T T T T .    
300	A A A A A .    
301	T T T T T .    
302	T T T T T .    
303	C C C C C .    
304	C C C C C .    
305	G G G G G .    
306	T T T T T .    
307	A A A A A .    
308	T T T T T .    
309	T T T T T .    
310	C C C C C .    
311	C C C C C .    
312	T T T T T .    
313	T T T T T .    
314	T T T T T .    
315	G G G G G .    
316	T T T T T .    
317	T T T T T .    
318	T T T T T .    
319	T T T T T .    
320	T T T T T .    
321	G G G G G .    
322	G G G G G .    
323	C C C C C .    
324	A A A A A .    
325	A A A A A .    
326	T T T T T .    
327	T T T T T .    
328	C C C C C .    
329	T T T T T .    
330	G G G G G .    
331	T T T T T .    
332	A A A A A .    
333	A A A A A .    
334	A A A A A .    
335	G G G G G .    
336	A A A A A .    
337	A A A A A .    
338	G G G G G .    
339	T T T T T .    
340	G G G G G .    
341	A A A A A .    
342	A A A A A .    
343	A A A A A .    
344	C C C C C .    
345	C C C C C .    
346	T T T T T .    
347	C C C C C .    
348	C C C C C .    
349	G G G G G .    
350	A A A A A .    
351	A A A A A .    
352	G G G G G .    
353	G G G G G .    
354	G G G G G .    
355	G G G G G .    
356	G G G G G .    
357	T T T T T .    
358	T T T T T .    
359	T T T T T .    
360	G G G G G .    
361	T T T T T .    
362	T T T T T .    
363	T T T T T .    
364	T T T T T .    
365	T T T T T .    
366	G G G G G .    
367	T T T T T .    
368	G G G G G .    
369	G G G G G .    
370	A A A A A .    
371	G G G G G .    
372	A A A A A .    
373	A A A A A .    
374	G G G G G .    
375	T T T T T .    
376	G G G G G .    
377	C C C C C .    
378	A A A A A .    
379	A A A A A .    
380	T T T T T .    
381	G G G G G .    
382	A A A A A .    
383	A A A A A .    
384	A A A A A .    
385	A A A A A .    
386	C C C C C .    
387	C C C C C .    
388	T T T T T .    
389	T T T T T .    
390	T T T T T .    
391	C C C C C .    
392	T T T T T .    
393	C C C C C .    
394	T T T T T .    
395	G G G G G .    
396	T T T T T .    
397	G G G G G .    
398	C C C C C .    
399	T T T T T .    
400	C C C C C .    
401	A A A A A .    
402	G G G G G .    
403	A A A A A .    
404	C C C C C .    
405	A A A A A .    
406	C C C C C .    
407	C C C C C .    
408	T T T T T .    
409	T T T T T .    
410	A A A A A .    
411	G G G G G .    
412	A A A A A .    
413	G G G G G .    
414	T T T T T .    
415	T T T T T .    
416	T T T T T .    
417	C C C C C .    
418	T T T T T .    
419	T T T T T .    
420	A A A A A .    
421	T T T T T .    
422	T T T T T .    
423	A A A A A .    
424	T T T T T .    
425	T T T T T .    
426	A A A A A .    
427	T T T T T .    
428	T T T T T .    
429	A A A A A .    
430	T T T T T .    
431	- T T - T .  2	 indel  
432	- A A - A .  2	 indel  
433	- T T - T .  2	 indel  
434	- T T - T .  2	 indel  
435	- A A - A .  2	 indel  
436	- T T - T .  2	 indel  
437	A A A A T .    
438	G G G G G .    
439	G G G G G .    
440	C C C C C .    
441	T T T T T .    
442	G G G G G .    
443	C C C C C .    
444	C C C C C .    
445	A A A A A .    
446	A A A A A .    
447	T T T T T .    
448	G G G G G .    
449	G G G G G .    
450	G G G G G .    
451	T T T T T .    
452	T T T T T .    
453	G G G G G .    
454	C C C C C .    
455	T T T T T .    
456	G G G G G .    
457	A A A A A .    
458	A A A A A .    
459	G G G G G .    
460	T T T T T .    
461	G G G G G .    
462	T T T T T .    
463	T T T T T .    
464	G G G G G .    
465	T T T T T .    
466	A A A A A .    
467	A A A A A .    
468	C C C C C .    
469	T T T T T .    
470	T T T T T .    
471	A A A A A .    
472	G G G G G .    
473	T T T T T .    
474	T T T T T .    
475	A A A A A .    
476	T T T T T .    
477	G G G G G .    
478	A A A A A .    
479	C C C C C .    
480	A A A A A .    
481	G G G G G .    
482	T T T T T .    
483	T T T T T .    
484	G G G G G .    
485	A A A A A .    
486	C C C C C .    
487	T T T T T .    
488	A A A A A .    
489	T T T T T .    
490	A A A A A .    
491	C C C C C .    
492	A A A A A .    
493	G G G G G .    
494	T T T T T .    
495	G G G G G .    
496	A A A A G .    
497	G G G G G .    
498	G G G G G .    
499	G G G G G .    
500	T T T T T .    
501	C C C C C .    
502	C C C C C .    
503	C C C C C .    
504	T T T T T .    
505	G G G G G .    
506	T T T T T .    
507	G G G G G .    
508	G G G G G .    
509	T T T T T .    
510	A A A A A .    
511	C C C C C C    
512	A A A A A A    
513	T T T T T T    
514	T T T T T T    
515	C C C C C C    
516	C C C C C C    
517	A A A A A A    
518	G G G G G G    
519	C C C C C C    
520	G G G G G G    
521	G G G G G G    
522	T T T T T T    
523	C C C C C C    
524	T T T T T T    
525	G G G G G G    
526	T T T T T T    
527	G G G G G G    
528	A A A A A A    
529	T T T T T T    
530	T T T T T T    
531	A A A A A A    
532	T T T T T T    
533	T T T T T T    
534	T T T T T T    
535	T T T T T T    
536	C C C C C C    
537	T T T T T T    
538	T T T T T T    
539	A A A A A A    
540	A A A A A A    
541	T T T T T T    
542	T T T T T T    
543	C C C C C C    
544	A A A A A A    
545	C C C C C C    
546	C T T T T T    
547	. T T T T T    
548	. C C C C C    
549	. T T T T T    
550	. G G G G G    
551	. A A A A A    
552	. A A A A A    
553	. T T T T T    
554	. T T T T T    
555	. C C C C C    
556	. C C C C C    
557	. T T T T T    
558	. G G G G G    
559	. G G G G G    
560	. T T T T T    
561	. T T T T T    
562	. T T T T T    
563	. G G G G G    
564	. T T T T T    
565	. G G G G G    
566	. T T T T T    
567	. G G G G G    
568	. T T T T T    
569	. C C C C C    
570	. T T T T T    
571	. A A A A A    
572	. C C C C C    
573	. G G G G G    
574	. T T T T T    
575	. T T T T T    
576	. G G G G G    
577	. G G G G G    
578	. G G G G G    
579	. T T T T T    
580	. T T T T T    
581	. G G G G G    
582	. T T T T T    
583	. G G G G G    
584	. T T T T T    
585	. A A A A A    
586	. C C C C C    
587	. A A A A A    
588	. C C C C C    
589	. A A A A A    
590	. C C C C C    
591	. A A A A A    
592	. C C C C C    
593	. T T T T T    
594	. T T T T T    
595	. G G G G G    
596	. C C C C C    
597	. G G G G G    
598	. T T T T T    
599	. G G G G G    
600	. T T T T T    
601	. A A A A A    
602	. A A A A A    
603	. G G G G G    
604	. A A A A A    
605	. G G G G G    
606	. T T T T T    
607	. T T T T T    
608	. T T T T T    
609	. C C C C C    
610	. A A A A A    
611	. T T T T T    
612	. T T T T T    
613	. T T T T T    
614	. T T T T T    
615	. C C C C C    
616	. A A A A A    
617	. C C C C C    
618	. T T T T T    
619	. G G G G G    
620	. G G G G G    
621	. T T T T T    
622	. A A A A A    
623	. A A A A A    
624	. A A A A A    
625	. T T T T T    
626	. G G G G G    
627	. G G G G G    
628	. T T T T T    
629	. T T T T T    
630	. G G G G G    
631	. A A A A A    
632	. A A A A A    
633	. C C C C C    
634	. A A A A A    
635	. T T T T T    
636	. A A A A A    
637	. G G G G G    
638	. G G G G G    
639	. T T T T T    
640	. T T T T T    
641	. G G G G G    
642	. C C C C C    
643	. T T T T T    
644	. G G G G G    
645	. T T T T T    
646	. G G G G G    
647	. G G G G G    
648	. T T T T T    
649	. G G G G G    
650	. G G G G G    
651	. G G G G G    
652	. A A A A A    
653	. T T T T T    
654	. T T T T T    
655	. G G G G G    
656	. A A A A A    
657	. T T T T T    
658	. T T T T T    
659	. G G G G G    
660	. C C C C C    
661	. A A A A A    
662	. T T T T T    
663	. A A A A A    
664	. G G G G G    
665	. A A A A A    
666	. T T T T T    
667	. A A A A A    
668	. A A A A A    
669	. T T T T T    
670	. A A A A A    
671	. A A A A A    
672	. C C C C C    
673	. T T T T T    
674	. C C C C C    
675	. G G G G G    
676	. C C C C C    
677	. C C C C C    
678	. T T T T T    
679	. C C C C C    
680	. A A A A A    
681	. T T T T T    
682	. A A A A A    
683	. T T T T T    
684	. C C C C C    
685	. T T T T T    
686	. T T T T T    
687	. T T T T T    
688	. T T T T T    
689	. C C C C C    
690	. C C C C C    
691	. C C C C C    
692	. C C C C C    
693	. A A A A A    
694	. A A A A A    
695	. A A A A A    
696	. A A A A A    
697	. C C C C C    
698	. A A A A A    
699	. A A A A A    
700	. T T T T T    
701	. A A A A A    
702	. A A A A A    
703	. T T T T T    
704	. A A A A A    
705	. G G G G G    
706	. G G G G G    
707	. A A A A A    
708	. G G G G G    
709	. T T T T T    
710	. T T T T T    
711	. A A A A A    
712	. A A A A A    
713	. T T T T T    
714	. C C C C C    
715	. A A A A A    
716	. A A A A A    
717	. C C C C C    
718	. T T T T T    
719	. G G G G G    
720	. C C C C C    
721	. T T T T T    
722	. G G G G G    
723	. T T T T T    
724	. T T T T T    
725	. T T T T T    
726	. T T T T T    
727	. T T T T T    
728	. C C C C C    
729	. A A A A A    
730	. A A A A A    
731	. T T T T T    
732	. G G G G G    
733	. A A A A A    
734	. C C C C C    
735	. G G G G G    
736	. A A A A A    
737	. G G G G G    
738	. A A A A A    
739	. G G G G G    
740	. A A A A A    
741	. G G G G G    
742	. A A A A A    
743	. G G G G G    
744	. A A A A A    
745	. G G G G G    
746	. T T T T T    
747	. C C C C C    
748	. A A A A A    
749	. T T T T T    
750	. A A A A A    
751	. G G G G G    
752	. T T T T T    
753	. G G G G G    
754	. A A A A A    
755	. T T T T T    
756	. G G G G G    
757	. A A A A A    
758	. T T T T T    
759	. G G G G G    
760	. A A A A A    
761	. T T T T T    
762	. C C C C C    
763	. T T T T T    
764	. T T T T T    
765	. T T T T T    
766	. T T T T T    
767	. G G G G G    
768	. . A A A A    
769	. . T T T T    
770	. . G G G G    
771	. . C C C C    
772	. . T T T T    
773	. . A A A A    
774	. . T T T T    
775	. . T T T T    
776	. . T T T T    
777	. . G G G G    
778	. . A A A A    
779	. . C C C C    
780	. . T T T T    
781	. . T T T T    
782	. . C C C C    
783	. . T T T T    
784	. . G G G G    
785	. . T T T T    
786	. . G G G G    
787	. . G G G G    
788	. . T T T T    
789	. . T T T T    
790	. . G G G G    
791	. . A A A A    
792	. . T T T T    
793	. . C C C C    
794	. . C C C C    
795	. . A A A A    
796	. . C C C C    
797	. . A A A A    
798	. . C C C C    
799	. . T T T T    
800	. . C C C C    
801	. . A A A A    
802	. . G G G G    
803	. . A A A A    
804	. . T T T T    
805	. . G G G G    
806	. . A A A A    
807	. . T T T T    
808	. . T T T T    
809	. . G G G G    
810	. . T T T T    
811	. . C C C C    
812	. . T T T T    
813	. . G G G G    
814	. . G G G G    
815	. . G G G G    
816	. . A A A A    
817	. . T T T T    
818	. . T T T T    
819	. . G G G G    
820	. . T T T T    
821	. . C C C C    
822	. . T T T T    
823	. . G G G G    
824	. . G G G G    
825	. . A A A A    
826	. . A A A A    
827	. . A A A A    
828	. . A A A A    
829	. . C C C C    
830	. . A A A A    
831	. . G G G G    
832	. . A A A A    
833	. . T T T T    
834	. . G G G G    
835	. . T T T T    
836	. . A A A A    
837	. . A A A A    
838	. . T T T T    
839	. . T T T T    
840	. . A A A A    
841	. . T T T T    
842	. . A A A A    
843	. . A A A A    
844	. . A A A A    
845	. . T T T T    
846	. . G G G G    
847	. . G G G G    
848	. . T T T T    
849	. . T T T T    
850	. . A A A A    
851	. . T T T T    
852	. . A A A A    
853	. . T T T T    
854	. . G G G G    
855	. . A A A A    
856	. . T T T T    
857	. . T T T T    
858	. . A A A A    
859	. . A A A A    
860	. . A A A A    
861	. . A A A A    
862	. . G G G G    
863	. . C C C C    
864	. . C C C C    
865	. . T T T T    
866	. . G G G G    
867	. . C C C C    
868	. . T T T T    
869	. . C C C C    
870	. . T T T T    
871	. . . . . A    
872	. . . . . T    
873	. . . . . C    
874	. . . . . T    
875	. . . . . T    
876	. . . . . G    
877	. . . . . T    
878	. . . . . G
```
